# Utility of Raman Spectroscopy in Pulmonary Medicine

**DOI:** 10.3390/arm92050038

**Published:** 2024-10-18

**Authors:** Pauls Dzelve, Arta Legzdiņa, Andra Krūmiņa, Madara Tirzīte

**Affiliations:** 1 Department of Internal Medicine, Faculty of Medicine, Riga Stradiņš University, LV1007 Riga, Latvia; arta.legzdina@rsu.lv (A.L.); andra.krumina@rsu.lv (A.K.); madara.tirzite@rsu.lv (M.T.); 2 Clinical Centre “Gaiļezers”, Riga East University Hospital, LV1038 Riga, Latvia

**Keywords:** pulmonology, saliva analysis, COPD, phenotyping, Raman spectroscopy, screening

## Abstract

**Highlights:**

**What are the main findings?**

- Usage of Raman spectroscopy in respiratory medicine is still in the early stages of development. Most studies have focused on malignant tissue identification and staging, with good results.
- Saliva analysis using Raman spectroscopy has great potential to successfully identify different COPD phenotypes, pathogens and malignant tissue, but needs further research.

**What are the implications of the main findings?**

- Raman spectroscopy can be used to successfully identify malignant tissue and help in cancer staging.
- Saliva analysis using Raman spectroscopy shows promise to improve COPD patient phenotyping. This helps to identify risk factors and reduce overall disease burden and hospitalization risk.

**Abstract:**

The Raman effect, or as per its original description, “modified scattering”, is an observation that the number of scattered light waves shifts after photons make nonelastic contact with a molecule. This effect allows Raman spectroscopy to be very useful in various fields. Although it is well known that Raman spectroscopy could be very beneficial in medicine as a diagnostic tool, there are not many applications of Raman spectroscopy in pulmonary medicine. Mostly tumor tissue, sputum and saliva have been used as material for analysis in respiratory medicine. Raman spectroscopy has shown promising results in malignancy recognition and even tumor staging. Saliva is a biological fluid that could be used as a reliable biomarker of the physiological state of the human body, and is easily acquired. Saliva analysis using Raman spectroscopy has the potential to be a relatively inexpensive and quick tool that could be used for diagnostic, screening and phenotyping purposes. Chronic obstructive pulmonary disease (COPD) is a growing cause of disability and death, and its phenotyping using saliva analysis via Raman spectroscopy has a great potential to be a dependable tool to, among other things, help reduce hospitalizations and disease burden. Although existing methods are effective and generally available, Raman spectroscopy has the benefit of being quick and noninvasive, potentially reducing healthcare costs and workload.

## 1. Introduction

The Raman effect, originally described as “modified scattering” and discovered by professor Sir Chandrasekhara Venkata Raman (7 November 1888–21 November 1970) alongside his student Kariamanikkam Srinivasa Krishnan (4 December 1898–14 June 1961) on 28th February 1928 in Calcutta, describes a previously unknown physical phenomenon that light, as it traverses material, changes its wavelength and frequency after nonelastic contact with a molecule [1]. This can otherwise be observed as light changing its color. This was, at the time, considered to be proof of quantum theory and that light has a quantum nature [2]. In 1930, after being nominated by Lord Rutherford, professor Sir C.V. Raman became the first person of Indian or non-white descent to receive the Nobel Prize in Physics for the aforementioned discovery [2].

Raman spectroscopy has since been used in a variety of applications, for example, the pharmaceutical industry and the development of new drugs, the development of polymer compounds, carbon materials including carbon nanotubes, semiconductors, and the identification of chemical and explosives. Recently, new studies have been published that expand the utility of Raman spectroscopy to the medical sciences. Most notably, recent publications have detailed its applications in forensic science [3], with the ability to detect illicit substances and successfully detect the smoking status of the subject [4,5]. Saliva can be used to test for the presence of illicit substances, including for anti-doping purposes, and has advantages such as quicker sampling and a reduced probability of adultery compared to other body fluids [6,7]. Recently, published studies have been conducted to find reliable disease biomarkers in tissue samples as well as body fluids such as urine, semen, cerebrospinal fluid, blood, saliva and sputum [6,8]. The most notable advances and studies have been in the medical fields including oncology, cardiovascular disease, the identification of pathogens such as SARS-CoV-2, hepatitis B virus [8] and influenza [9,10] along with other viruses and bacteria, neurodegenerative disease including Parkinson’s disease and Alzheimer’s [6,8,11,12,13], the development of new biomarkers and the development of various risk stratification tools. Regarding the identification of malignant tissues, Raman spectroscopy has been investigated in prostate [8], lung [8,14,15,16,17,18,19,20,21,22,23,24], breast, skin, colorectal, cervical and other tissue [8]. It is certainly worth mentioning that the ability of Raman spectroscopy to reliably distinguish between benign and malignant tissues [8,14,15,16,17] means that it could have an important role in cancer staging [23]. The use of this method as a quick tool to evaluate tissue during surgery promises certain advantages discussed later in this publication [14].

The detection of cardiovascular and neurodegenerative disease markers such as troponin, NT-proBNP, Aβ and others has been analyzed numerous studies [8]. Regarding pulmonary medicine, most studies have focused on malignancy biomarkers and, more recently due to pandemic, the detection of SARS-CoV-2 and the monitoring of the spread of diseases [8,9,10,25,26].

As a leading cause of death and disability worldwide [27], chronic obstructive pulmonary disease (COPD) has severe quality of life and socioeconomic impacts. New disease biomarkers could prove beneficial in disease phenotyping and control. Potential advantages include relatively simple preparation of the sample, the sample is preserved instead of being destroyed, relatively low cost and quick measurement. This makes biomarker testing using saliva analysis via Raman spectroscopy to phenotype disease a good tool that can be implemented in an outpatient setting, thus helping to improve disease management.

The aim of this review is to investigate the role of saliva analysis using Raman spectroscopy in the respiratory medicine field.

## 2. Utility of Raman Spectroscopy in Pulmonary Medicine

Regarding pulmonary medicine, the most commonly used biological materials that are analyzed are saliva and lung tissue samples taken during thoracic surgery. The latter has the potential to be used during surgery, thus reducing length of procedure, decreasing biopsy count, and potentially producing higher yields. The oncology field is being extensively studied. One study by Bourbousson et al. in 2019 successfully identified differences in the characteristics of tumorous and nontumorous lung tissue; it was revealed that Raman spectroscopy can successfully identify tumor tissue even when the tissue sample was visually uniform after hemotoxylin and eosin staining. [14] This shows that spectroscopic molecular analysis may provide a more objective diagnosis than morphology-based imaging [14]. Another study by Zheng et al. in 2019 analyzed biomolecular changes in lung cancer and successfully applied Raman spectroscopy to achieve a cytopathological diagnosis with an accuracy of almost 99% [15]. A promising prospect is the use of Raman spectroscopy to characterize solitary pulmonary nodules in vivo [16,17]. In 2017, McGregor et al. used a real-time endoscopic Raman spectroscopy system to aid specificity in autofluorescence bronchoscopy in combination with white light bronchoscopy. Eighty patients were included, and clinical data from 280 tissue sites were included, 72 of which were high-grade dysplasias or malignancies [16,17]. The data from this study show high sensitivity (90%) and good specificity (65%).

In a meta-analysis performed by Chen et al. in 2021 that included 12 studies, the pooled diagnostic sensitivity of Raman spectroscopy in lung cancer was 0.90 (95% CI, 0.87–0.92, *p* < 0.05), and the specificity was 0.76 (95% CI, 0.72–0.79, *p* < 0.05). The pooled positive likelihood ratio was 5.87 (95% CI, 3.45–9.97), and the negative likelihood ratio was 0.14 (95% CI, 0.10–0.22). These data show that the use of Raman spectroscopy in lung cancer diagnostics is of high sensitivity and considerable specificity [24].

Serum biomarker analysis using Raman spectroscopy in relation to lung disease has also been proposed. A study by Wang et al. in 2018 [23] investigated the cost-effectiveness of serum laser Raman spectroscopy in the screening and staging of non-small cell lung cancer (NSCLC). Peripheral venous blood was analyzed in 91 subjects in five groups—healthy control group, stage I NSCLC, stage II NSCLC, stage III NSCLC and stage IV NSCLC. The overall accuracy rate was 92%, the sensitivity was 86% in healthy subjects, 65% in stage I NSCLC, 75% in stage II NSCLC and 87% in stage III/IV NSCLC, and the specificity was 95%, 94%, 88% and 93%, respectively [23]. In another study of the diagnosis of COVID-19 in the blood serum of 10 healthy and 10 COVID-19-positive patients, RT-PCR RNA and ELISA tests were analyzed. The results showed a sensitivity of 87% and a specificity of 100%, which shows the rapid and cost-effective nature of the method in diagnosing COVID-19 [28].

Analyses of light-colored samples with other techniques such as Fourier transform infrared spectroscopy (FTIR), which is mainly spectroscopic, and coherent anti-Stokes Raman spectroscopy (CARS), which is mainly used in imaging, for the diagnosis and staging of lung cancer has been studied over the last decade and has increased in significance [18]. Both FTIR and Raman spectroscopy are vibrational spectroscopy techniques with certain differences in how the molecules are excited and how the measurements are made. An advantage of Raman spectroscopy over FTIR is that the former requires less material preparation. Coherent anti-Stokes Raman spectroscopy uses multiple photons and is thus stronger than Raman emission. These techniques have been used in various studies that investigated the diagnostic potential of lung cancer and have an accuracy of up to 99% [19,20,21,22]. The FTIR method has also been investigated in analyses of biochemical profiles of sputum and revealed noticeable differences between sputa from healthy subjects and those with COPD, although the drawbacks of the study included an age mismatch between the groups, and no group included smokers with normal lung function [5].

## 3. Saliva Analysis in Pulmonary Medicine

Saliva analysis has been proposed as a beneficial method to identify various diseases. It is a biological fluid with an increasing diagnostic and prognostic importance and, with its variability, is thought to be a reliable indicator of the physiological state of the human body, including hormonal, emotional, nutritional and metabolic variations [29,30,31]. It is also worth noting that salivary composition has both glandular and non-glandular origins. It is composed mostly of oral mucosal transudate, upper airway secretions, bacteria, fungi, viruses and crevicular fluid, which in some respects can be considered a plasma transudate [9,31,32]. The collection of saliva samples is relatively easy, with few contraindications. It is nontraumatic, painless and quick, which is why it is perceived favorably by patients. Also, it requires minimal operator training and is relatively cost-effective. This makes it a promising area for the research and development of future methods, especially when discussing disease screening and phenotyping in early disease stages. Still, there is a need to differentiate between stimulated and unstimulated (or basal drool) saliva. Although very similar, stimulated and unstimulated saliva can differ when using Raman spectroscopy analysis; thus, a standardized collection protocol is advised to be implemented whenever salivary analysis is performed [25]. Also, one should bear in mind that the salivary composition can change not only due to various diseases but also according to the circadian rhythm [33].

Although studied in other applications such as illicit drug detection, forensics, dentistry, viral and bacterial strain detection, and dentistry [33], currently, the use of saliva analysis in the pulmonology field is still in its early stages of development [34]. Nevertheless, it has been studied in several respiratory diseases, often in correlation with cytokines and inflammatory proteins, oxidative-stress-related biomarkers and tumor markers. Also, various causative agents can be found in saliva in the setting of infectious diseases such as influenza and SARS-CoV-2, which could be helpful in certain patient cases [9,10]. In a 2022 study by Melo-Dias et al. comprising 128 individuals, saliva was investigated as a noninvasive specimen for COPD assessment by profiling the 16S rRNA of oral bacteria in 70 COPD patients and 58 controls. The clustering analysis separated patients into two groups based on microbiota composition, and these groups differed in hospitalization rates due to COPD exacerbation and the number of GOLD class D patients. Furthermore, a low frequency of *Prevotella* was associated with a significantly higher risk of severe exacerbation and being GOLD class D, with a loss of microbiota diversity and an increase in *Proteobacteria*, especially *Haemophilus*, and *Streptococcus* was seen in COPD, although there was a poor association with clinical features [35]. In a 2016 study, salivary MMP-9 activity was compared with FVC, FEV1 and FEV1/FVC in 30 patients to investigate whether pulmonary function tests could be used to estimate MMP-9 activity. Unfortunately, the results came out statistically insignificant [36], and this area requires more research.

In a 2018 study by Qian et al., the ability of a saliva test to detect lung cancer using Raman spectroscopy was investigated. Included in this study were 61 lung cancer patients and 66 healthy controls. The results were promising, with the leave-one-out algorithm producing a sensitivity of 95% and a specificity of 100% and the random forest algorithm yielding a sensitivity of 96.7% and a specificity of 100% [37].

As a noninvasive tool, saliva analysis using Raman spectroscopy could become beneficial for recognizing different phenotypes and risk factors of certain diseases early in their courses. A great example of this principle is a study by Rusciano et al. in 2013 that used Raman spectroscopy to detect bacteria in the sputum of patients with cystic fibrosis [10]. Common disease-causing agents such as *Pseudomonas aeruginosa* and *Staphylococcus aureus*, among others, were identified correctly, with a global accuracy of more than 95% [32]. These results promise a faster, more cost-effective and timelier identification of such bacteria with no need for bacterial isolation or cultures, and, in turn, enable timelier therapy to achieve a lower disease burden. In another study, surface-enhanced Raman spectroscopy was used to identify correlation in salivary findings and lung cancer, with an accuracy, sensitivity and specificity of 80%, 78% and 83%, respectively [38]. This could allow earlier and more precise diagnostics and screening, which, in turn, can lead to improved short- and long-term results and increase survival.

Raman spectroscopy analysis combined with two-dimensional shear wave elastography could aid clinicians in the diagnosis of Sjögren’s syndrome. In a study published in 2020, 31 patients with primary Sjögren’s syndrome, according to the ACR/EULAR classification criteria, were included. The overall classification accuracy was 81% [39].

In a study published in 2019 by Zamora-Mendoza et al., 44 subjects aged 6–12 years with a diagnosis of asthma were included. An immunoassay was used for the quantification of 37 cytokines in the subjects’ saliva, and an analysis using surface-enhanced Raman spectroscopy with a Raman microscope was performed. There was a significant association between IL-8, IL-10 and sCD163. The Raman spectra showed significant amplification in the region of 760 to 1750 cm^−1^. The previously described principal component analysis and linear discriminant analysis (PCA-LDA) method had a sensitivity of 85%, a specificity of 82% and an accuracy of 84% for the diagnosis of asthma [40].

COPD is defined by the 2024 GOLD guidelines as a heterogeneous lung condition characterized by chronic respiratory symptoms due to abnormalities of the airways and alveoli that cause persistent, often progressive, airflow obstruction. It is currently a leading cause of mortality and morbidity worldwide. COPD is a major health concern due to its wide array of associated comorbidities such as cardiovascular disease, malignancy, sleep disorders and others, and often involves a multidisciplinary approach that includes multiple specialists. The majority of COPD cases are considered to be caused by environmental factors, namely tobacco smoking and pollution, which are potentially preventable, and, although the disease itself is irreversible, the symptoms of COPD are treatable [27].

In a pilot study published in 2021 by Carlomagno et al., 15 COPD patients and 15 healthy patients were included. The researchers found that Raman analysis provided an opportunity to recognize specific signatures in COPD patients’ saliva that have been previously studied and characterized. A classification model that arose from this has an accuracy, specificity and sensitivity of more than 98% [41]. This expands the possibilities for future research. The ability to identify specific signatures in a patient’s saliva enables determinations of disease phenotypes, further disease progression and exacerbation risk. This can lead to a more effective treatment that is customized for each individual.

The authors of this publication take part in the ERA-Net PerMed multicentric project ”Raman analysis of saliva from COPD patients as new biomarker: AI-based point-of-care for the disease monitoring and management”, which takes a deeper look at COPD phenotyping using Raman spectroscopy to analyze saliva to identify biomarkers, and the analyzed biomarkers have been discussed in the main part of this article. As the progress in disease management and diagnostics is advancing, there is an increasing need to identify certain at-risk groups early in the course of the disease and coordinate therapy accordingly to successfully lower disease progression and the risk of complications. COPD that is better controlled leads to lower economic, social and health-related disease burdens and decreases hospitalizations. The aforementioned study is currently ongoing, and the results could lead to promising new insights into COPD risk stratification and patient profiling.

## 4. Discussion

Although studies regarding the use of Raman spectroscopy in respiratory medicine are scarce, one can identify several potential utilities—the differentiation of pulmonary nodules, lung cancer and different phenotypes of various chronic lung diseases. As disease management and treatment are increasingly driven by a personalized approach, depending on the disease’s phenotypic, endotypic, genetic and regiotypic characteristics as well as a person’s individual traits, a method capable of include different variables in the assessment is highly valuable. An early identification of a disease and its characteristics could be beneficial in risk-reduction strategies, possibly enabling earlier treatment and lifestyle modification.

Saliva, as a biological material, is a source of abundant information that not only contains a vast spectrum of biological substances but also various microorganisms and provides information about a patient’s state and their individual variations as well as characteristics of the disease. The collection of saliva samples is relatively easy, is well tolerated by patients, has a low cost, is fast and requires little operator training. A standard protocol should be included to simplify the collection instructions and reduce the error rate. As a cost-effective and quick tool, it could introduce better screening, which, in turn, could be used to identify possible disease traits early and reduce the overall disease and financial burdens.

Current technical limitations include limited availability of Raman spectroscopy equipment in most medical centres outside of scientific institutions and the possibility of incorrect sample collection and storage. Although there are a lot of studies and possible applications, the routine use of Raman spectroscopy in the medical field is currently not commonplace but is expected to increase in the future. Moreover, as this method is expected to be used not only a diagnostic tool but also as a screening tool, some transportation challenges could arise for outpatient clinics. It is worth noting that sample storage is relatively simple and does not require overly specific or expensive equipment. It is beneficial if the saliva is stored at −20 °C, with a minimal storage time, a lower sample volume and an enzyme inhibitor, unless the sample is analyzed soon after collection [39].

Although shown to be very precise, rapid and sensitive in the detection of biomarkers even at low concentrations, Raman spectroscopy, as of now, needs specific protocols and advanced analyses of the results, including mathematical analysis and machine learning [32], which are being investigated in ongoing studies. Also, it must be noted that saliva analysis using Raman spectroscopy in respiratory medicine should be used in conjunction with other diagnostic methods, including pulmonary function tests, imaging results and others to provide a broader context, and is currently not a standalone method.

## 5. Conclusions and Future Directions

Future research that aids in finding the best utility of Raman spectroscopy in pulmonary medicine should aim not only for distinctions between different disease traits but also for close patient matching in the studied groups, taking into account various extrinsic and intrinsic variations. Saliva analysis using Raman spectroscopy has early diagnostic potential, could be included in screening and phenotyping programs for various malignant and nonmalignant diseases, and should be investigated further.

## Data Availability

No new data were created or analyzed in this study. Data sharing is not applicable to this article.
